# CCAAT/Enhancer-Binding Protein-α Suppresses Lung Tumor Development in Mice through the p38α MAP Kinase Pathway

**DOI:** 10.1371/journal.pone.0057013

**Published:** 2013-02-20

**Authors:** Atsuyasu Sato, Norishige Yamada, Yuya Ogawa, Machiko Ikegami

**Affiliations:** 1 Division of Pulmonary Biology, Cincinnati Children's Hospital Medical Center, University of Cincinnati, Cincinnati, Ohio, United States of America; 2 Division of Reproductive Sciences, Cincinnati Children's Hospital Medical Center, University of Cincinnati, Cincinnati, Ohio, United States of America; University of Giessen Lung Center, Germany

## Abstract

The transcription factor CCAAT/enhancer-binding protein α (C/EBPα) is a basic leucine zipper transcription factor and is expressed in alveolar type II cells, alveolar macrophages and Clara cells in the lung. Although decrease or absence of C/EBPα expression in human non-small cell lung cancer suggests a possible role of C/EBPα as a lung tumor suppressor, there is no direct proof for this hypothesis. In this study, we investigated, for the first time, the role of C/EBPα in lung tumors in vivo using transgenic mice with lung epithelial specific conditional deletion of *Cebpa* (*Cebpα^Δ/Δ^* mice) and a urethane-induced lung tumor model. C/EBPα expression in the lung was dispensable, and its deletion was not oncogenic under unstressed conditions. However, at 28 wk after urethane injection, the number and size of tumors and the tumor burden were significantly higher in *Cebpα^Δ/Δ^* mice than in littermate control mice. Urethane-injected *Cebpα^Δ/Δ^* mice showed highly proliferative adenomas and adenocarcinomas in the lung, and survival time after urethane-injection was significantly shorter than that in control mice. In control mice, C/EBPα was strongly induced in the tumor tissues at 28 weeks after urethane-injection, but became weakened or absent as tumors progressed after long-term observation for over 1 year. Using intraperitoneal injection of p38 inhibitor (SB203580), we demonstrated that the induction of C/EBPα is strongly regulated by the p38 MAP kinase in murine alveolar epithelial cells. A high correlation was demonstrated between the expression of C/EBPα and p38α MAP kinase in tumor cells, suggesting that C/EBPα silencing in tumor cells is caused by down-regulation of p38α MAP kinase. In conclusion, the role of C/EBPα as a lung tumor suppressor was demonstrated for the first time in the present study, and the extinguished C/EBPα expression through p38α inactivation leads tumor promotion and progression.

## Introduction

Exposure to carcinogens, including ionizing radiation, viral infection, and tobacco smoking causes cumulative changes in the DNA of lung tissue [1] and initiates lung cancer by activating oncogenes and/or inactivating tumor suppressor genes [2]. CCAAT/enhancer-binding protein-α (C/EBPα) is a basic leucine zipper transcription factor that is expressed in many tissues [3]. C/EBPα plays an important role in normal tissue development, namely, in the regulation of cell proliferation and cell differentiation [4], [5].

In the lung, C/EBPα is expressed in alveolar type II cells (ATII cells), Clara cells, and alveolar macrophages from late gestation through adulthood [6]–[9]. The mechanisms that regulate lung epithelial development are often linked to lung injury-repair processes and lung disease. Experiments in transgenic mice with lung epithelial specific conditional deletion of C/EBPα (*Cebpα^Δ/Δ^* mice) have proved that C/EBPα is required for pulmonary maturation in late gestation [7] and potentially plays an emergent role to maintain lung homeostasis following lung injury and repair in adult mice [8], [9].

Previous studies have demonstrated decreased or absent of C/EBPα expression in 50% of stage II and IIIA lung adenocarcinomas, suggesting that C/EBPα may function as a tumor suppressor in the lung [10]–[12]. However, direct evidence for this hypothesis is lacking, and the mechanism underlying the decrease in C/EBPα expression in lung adenocarcinoma has not been well studied. Unlike hematopoietic malignancies, in which the CEBPA gene has been demonstrated to be mutated [13], CEBPA mutations are rare in non-small cell lung cancer (NSCLC) [14]. Absence of C/EBPα by the aberrant DNA methylation of C/EBPα promoter has been observed *in vitro* in human lung cancer cell lines and in human lung cancer tissues [12]. In addition, down-regulation of C/EBPα by highly expressed tribbles homolog2 (TRIB2) has been shown in lung cancer cell lines [15]. In animal model, *MAP kinase14^Δ/Δ^Kras^G12V^* mice with extensive lung stem cell numbers and tumor progression have demonstrated decreased C/EBPα expression *in vivo*, suggesting that C/EBPα is a possible downstream target of MAPK14 (p38α) in the lung [16].

In the present study, we tested the hypothesis that C/EBPα plays a role in the suppression of lung tumorigenesis *in vivo*. The deletion of C/EBPα in *Cebpα^Δ/Δ^* mice strikingly increased the urethane-induced lung tumor incidence and the malignant tumors. Furthermore, we demonstrated that the expression of p38α mitogen activated protein kinase (p38α MAP kinase) is required for C/EBPα expression in the lung.

## Materials and Methods

This study was carried out in strict accordance with the recommendations in the Guide for the Care and Use of Laboratory Animals of the National Institutes of Health. All study protocols were approved by the Institutional Animal Care and Use Committee of the Cincinnati Children's Hospital Research Foundation (Permit Number: 9D05044). All surgery was performed under sodium pentobarbital anesthesia, and all efforts were made to minimize suffering.

### Animals

Transgenic mice with lung specific conditional deletion of C/EBPα were developed previously [8] and were maintained in a mixed FVB/N and C57/BL6 genetic background. Briefly, the *Scgb1a1-rtTA^−/tg^*-line-1 mice were bred with *(tetO)_7_CMV-Cre^tg/tg^*/*Cebpa^flox/flox^* mice to produce *Scgb1a1-rtTA^−/tg^*/*(tetO)_7_CMV-Cre^−/tg^*/*Cebpa^+/flox^* mice. These mice were bred with *(tetO)_7_CMV-Cre^tg/tg^*/*Cebpa^flox/flox^* mice to generate triple transgenic *Scgb1a1-rtTA^−/tg^*/*(tetO)_7_CMV-Cre^tg/tg^*/*Cebpa^flox/flox^* mice (herein termed *Cebpα^Δ/Δ^* mice). The littermate *(tetO)_7_CMV-Cre^tg/tg^*/*Cebpa^flox/flox^* mice were used as controls.

Doxycycline (625 mg/kg; Harlan Teklad, Madison, WI) administered in the chow to the dams from embryonic day (E)0 to postnatal day (P)14 (the early-deletion study), provided sufficient doxycycline to the newborns through the milk (Figure S1A).

For the late-deletion study, doxycycline-containing chow was given to *Cebpα^Δ/Δ^* mice for 3 wk beginning at 14 wk of age, which was 8 to 11 wk after urethane injection. The comparison group of *Cebpα^Δ/Δ^* mice was given no-doxycycline containing normal food (No-Dox group).

### Tumor Induction by Urethane

For both the early-deletion and late-deletion studies, 1 mg/g body weight of urethane (Sigma, St. Louis, MO) dissolved in 0.9% NaCl (0.1 ml/g) was intraperitoneally injected to 6 week old mice twice. The second dose was injected 3 days after the first injection. The general condition of each mouse was monitored daily and the body weight was recorded weekly. Mice were euthanized at 10, 20, and 28 wk after the first urethane injection. In the long term study, control mice were euthanized when they lost more than 10% of their body weight at 6 wk of age.

The tumors on the lung surface were counted and diameter (r) was measured using microcallipers under a dissecting microscope [17]. The volume of tumor was calculated as T(n) = 3/4πr_(n)_^3^, where (n) is the number of tumors in the mouse and the tumor burden was calculated as 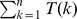. Fixed lung tumor sections were stained by haematoxylin and eosin (H&E) and were histologically classified according to the previously recommended criteria [18].

### Immunohistochemical and Immunofluorescence Analysis

Lungs were inflation fixed at 25 cmH_2_O and tissue sections were immuno-stained with anti-mouse C/EBPα, CC10, phospho-histone 3 (Santa Cruz Biotechnology, Santa Cruz, CA), cleaved caspase-3, p38 (Cell Signaling, Danvers, MA), Ki-67 (Dako, Carpinteria, CA), FoxA2, proSP-C (Sevenhills, Cincinnati, OH), 5-methyl cytosine (5-mC, EPIGENTEK, Farmingdale, NY) and negative control serum with or without microwave-citrate antigen retrieval. For dual labeling immunofluorescence, fluorescence was developed with Alex Fluor 488 and Alexa Fluor 594 conjugated secondary antiboies (Invitrogen, Carlsbad, CA). Tissue sections were stained and mounted in DAPI containing medium, (Invitrogen). The Zeiss Axioplan 2 microscope equipped with AxioVision software was utilized for microphotographs.

Mitotic index was calculated to evaluate cell proliferation in tumors. Mitotic index (%) = Ki-67 positive cells/total cells ×100.

TUNEL staining was performed using a TumorTACS™ In Situ Apoptosis Detection Kit (TREVIGEN, Gaithersburg, MD) according to the manufacturer's protocol.

### Real-time PCR

Homogenized left lung lobes were lysed in buffer and total RNA was isolated using an RNeasy Plus Mini-Kit (QIAGEN, Valencia, CA). Selected tumor samples on slide from paraffin embedded sections were isolated and RNA extraction was performed using the FFPE RNeasy-kit (Invitrogen). One microgram of total RNA was reverse transcribed to cDNA using Superscript VILO (Invitrogen). Quantitative RT-PCR for *Cebpa* (Mm00514283_m1), *Foxa2* (Mm00839704_mH), *Trib2* (Mm00454876_m1), *Spink5* (Mm_00511522_m1) and *Mapk14* (Mm00442497_m1) mRNA were performed using TaqMan® Gene Expression Assays (Applied Biosystems, Foster City, CA).

### Administration of p38 MAP Kinase Inhibitor

Control mice (6 wk of age) were injected intraperitoneally with p38 MAP kinase inhibitor, SB203580 (1 µM/kg, LC Laboratories, Woburn, MO), or 0.9% NaCl 3 times, at 12 hours intervals. The mice were euthanized for analysis 3 hours after last injection.

### DNA Methylation Analysis

All tumors were identified according to C/EBPα expression by immunohistochemistry. DNA and RNA analysis was performed on material isolated by hand under a dissection microscope from 4 serial slides for each tumor. DNA was isolated from each single tumor on paraffin sections using the FitAmp™ Paraffin Tissue Section DNA Isolation Kit (EPIGENTEK). For promoter methylation analysis, bisulfite modification was performed using Bisulflash™ DNA Modification Kit (EPIGENTEK). A DNA fragment in the C/EBPα promoter region was amplified by PCR using TaqGold PCR kit (Applied Biosystems) with primers designed by the web-based program MethPrimer (http://www.urogene.org/methprimer/index.html) (primer sequences: −337to−140: 5′GTTTTGGAAAGTTATAGGAGAAGG, 3′CACCCAATACCCCAACTAACTC, −140to+49: 5′TTTTTAGTGTTGGTTGGAAGTG, 3′CCTTCTCCTATAACTTTCC). The obtained DNA fragments were ligated with pGEM-T vector (Promega, Madison, WI) and transfected into Escherichia coli. Plasmid DNA purified from each colony was submitted to the Cincinnati Children's Hospital Medical Center DNA Sequence Core Unit for sequencing.

### Alveolar type II cell isolation

Alveolar type II cells were isolated from lungs using collagenase as previously reported [8].

### Statistical Analysis

Data were expressed as the means ± SEM. Statistical analyses were performed using GraphPad Prism 5.0c (GraphPad Software, La Jolla, CA). Comparisons between two groups were evaluated by *Mann-Whitney U test*. Non-parametric correlation analysis was performed using *Spearman's correlation test*. For multiple comparisons, analysis of variance (ANOVA) was used, followed by *Bonferroni's post hoc test* for significance. *Kaplan-Meier survival analysis* was used to compare the lifespans between groups.

## Results

### Deletion of C/EBPα in the Lung is not Oncogenic in Mice

In the present study, the *Scgb1a1-rtTA* promoter was used to induce lung epithelial specific deletion of C/EBPα. *Scgb1a1* is expressed in Clara cells from E16–E17 of gestation, and in ATII cells after birth. Thus, using this *Scgb1a1-rtTA* system for *Cebpα^Δ/Δ^* mice, the deletion of C/EBPα occurred in these *Scgb1a1*-expressing cells (Figure S1B). Lung structure was normal and no cell hyper-proliferation was observed in the lungs of *Cebpα^Δ/Δ^* mice as reported previously [9]. C/EBPα was clearly deleted from ATII cells at 6 wk of age.

Cancer development occurs in three major stages: initiation, promotion, and progression. To evaluate whether the absence of C/EBPα initiates tumor or not, spontaneous lung tumor in lung was observed up to 18 mo of age. We demonstrated that spontaneous lung tumors were not detected at 4 and 8.5 mo of age in both control and *Cebpα^Δ/Δ^* mice (n = 6/group). All mice enrolled in 18 months study (n = 25/group) survived for 18 months and spontaneous lung tumors were detected in 4 out of 25 control mice (16%) and 5 out of 25 *Cebpα^Δ/Δ^* mice (20%). The incidence of spontaneous lung tumors in this study was similar to that in previous reported study using FVB/N mice (13% at 14 mo of age and 41% at 24 mo) [19]. Thus, the deletion of C/EBPα in the lung epithelial cells did not alter the incidence of spontaneous lung tumors, demonstrating that the deletion of C/EBPα in the lung is not oncogenic in mice.

### Urethane-induced Lung Tumor Development is Significantly Increased in *Cebpα^Δ/Δ^* Mice

**Figure 1:**
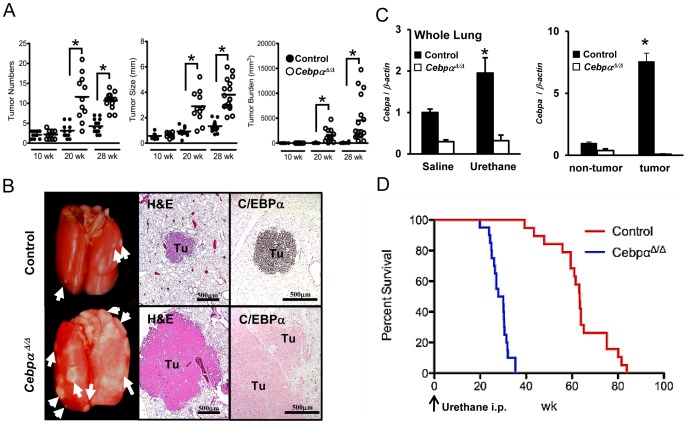
Urethane Induced Tumors in Early *Cebpa* Deletion Study. (A): The tumor numbers, tumor size and tumor burden were all significantly higher in the lungs of *Cebpα^Δ/Δ^* mice at 20 and 28 wk after urethane injection (10 wk: n = 10, 20 wk: n = 11, 28 wk: n = 17/group, *p<0.01). The bar indicates the average. (B): Appearance of the lung at 28 weeks after urethane injection. *Arrow*: surface tumors. Urethane injection induced lung tumors in both control and *Cebpα^Δ/Δ^* mice. Small tumors were observed on the surface of the lung in control mice. In contrast, *Cebpα^Δ/Δ^* mice developed multiple large tumors. Representative microphotographs of H&E stained sections showed the histology of the adenoma in control mice and adenocarcinomas with airway invasion in *Cebpα^Δ/Δ^* mice. Immunohistochemistry showed strong C/EBPα expression in tumor of control mice, but no staining in *Cebpα^Δ/Δ^* mice. Tu: Tumor. (C): *Cebpa* mRNA expression was evaluated by qRT-PCR. At 28 wk after urethane injection, *Cebpa* mRNA expression in control mice was significantly higher than saline injected mice (n = 4/group, p<0.05). The expression in *Cebpα^Δ/Δ^* mice was not affected by urethane injection. In control mice, *Cebpa* mRNA expression was induced in tumor tissues and was significantly higher than that in non-tumor lung tissues (n = 4/group, p<0.05). (D): *Kaplan-Meier* survival curves of control and *Cebpα^Δ/Δ^* mice after urethane injection (n = 20/group). *Cebpα^Δ/Δ^* mice showed significantly shorter survival than did control mice (log rank test, p<0.00001, the average survival after urethane injection: control mice (63.5±2.7 wk), *Cebpα^Δ/Δ^* mice (28.5±0.9 wk).

To investigate the role of C/EBPα in primary lung tumor promotion and progression, the lung tumor inducer urethane was injected into both control and *Cebpα^Δ/Δ^* mice at 6 wk of age. Three parameters for tumor enumeration including the number of tumors on the lung surface, the average diameter and the tumor burden were evaluated at 10, 20, and 28 wk after urethane injection (n = 10–17/group). All three parameters were similar at 10 wk, but significantly higher in *Cebpα^Δ/Δ^* mice at 20 and 28 wk after urethane injection compared to control mice (p<0.01) (Figure 1A). The tumors grew from 20 to 28 wk by merging with neighboring tumors, which resulted in larger tumor sizes and greater tumor burden, but fewer individual tumors at 28 wk compared to 20 wk after urethane injection. As shown in the representative photographs, lung tumors were larger and more numerous in the lung of *Cebpα^Δ/Δ^* mice compared to control mice 28 wk after urethane injection (Figure 1B, left column). All of the tumors in control mice were histologically classified as adenomas at 28 wk after urethane injection by H&E staining. The tumors over 5 mm in diameter that invades at least one large airways or the pleura were diagnosed as adenocarcinomas [18]. In *Cebpα^Δ/Δ^* mice, at least one adenocarcinoma was detected on each of the H&E stained slides (Figure 1B, middle column). Urethane-induced tumors in control mice at 28 wk after injection showed strong expression of C/EBPα protein (Figure 1B, right column) and *Cebpa* mRNA was significantly increased in urethane-injected control mice (Figure 1C). Furthermore, *Cebpa* mRNA expression was enriched in tumor tissues compared to non-tumor lung tissues in control mice (Figure 1C). The expression of *Cebpa* mRNA remained low in *Cebpα^Δ/Δ^* mice after urethane injection (Figure 1C). In survival study, *Cebpα^Δ/Δ^* mice showed significantly shorter survival than control mice after urethane injection (control mice: 63.5±2.7 wk, *Cebpα^Δ/Δ^* mice: 28.5±0.9 wk, p<0.00001, n = 20/group) (Figure 1D).

**Figure 2:**
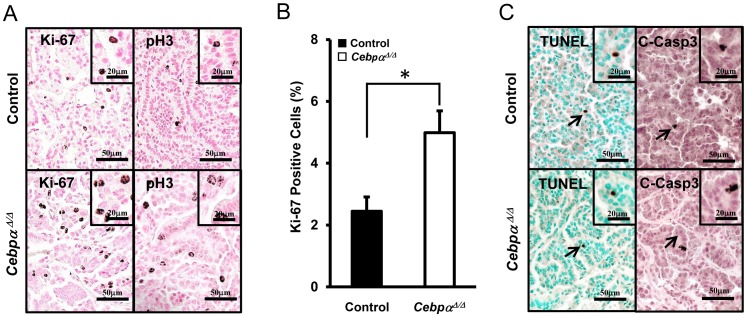
Cell Death and Proliferation in Urethane-Induced Tumors. (A): Immunohistochemistry for Ki-67 and pH 3 at 20 wk after urethane injection. (B): Mitotic index in the tumors of control and *Cebpα^Δ/Δ^* mice (n = 7/group). *Cebpα^Δ/Δ^* mice showed significantly higher numbers of Ki-67 positive cells in tumors (*p<0.01). The tumors in *Cebpα^Δ/Δ^* mice were more proliferative than those in control mice. (C): Arrows indicate TUNEL stain and cleaved-caspase 3 positive cells in tumors. Apoptotic cells were not obvious in this model.

The proliferation of lung tumor cells was evaluated by immunohistochemistry for Ki-67 and the mitosis-specific marker phospho-histone 3 (pH 3). Both markers revealed more mitotic cells in the tumors in *Cebpα^Δ/Δ^* mice than in control mice (Figure 2A). Mitotic index in the tumors was significantly higher in *Cebpα^Δ/Δ^* mice than in control mice at 20 wk after urethane injection, suggesting that the deletion of C/EBPα enhanced tumor proliferation (Figure 2B). As in the previous study of urethane-induced tumor model [20], apoptotic cells as evaluated by TUNEL and cleaved caspase-3 staining was rare and was not strongly detected in either control or *Cebpα^Δ/Δ^* mice. Neither TUNEL nor cleaved caspase-3 staining was affected by the deletion of C/EBPα (Figure 2C). Therefore, the deletion of C/EBPα affected tumor cell development primarily by enhancing cell proliferation.

### Promotion and Progression of Lung Tumor Stimulated by the Late-Deletion of *Cebpa*

**Figure 3:**
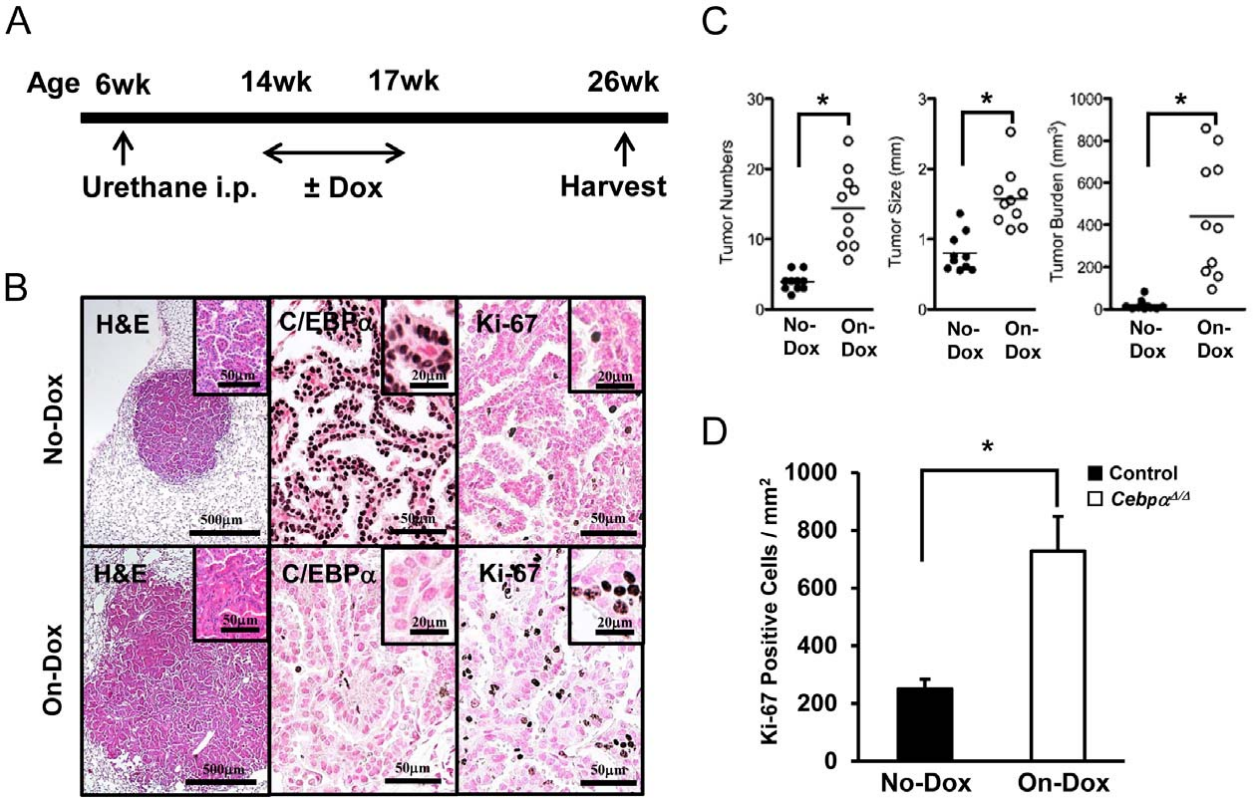
Late *Cebpa* Deletion Study. (A): Time course of urethane injection and doxycycline administration. Doxycycline-containing chow (on-Dox) or normal chow (no-Dox) was administered to *Cebpα^flox/flox^* mice for 3 weeks, beginning at 8 weeks after urethane injection. The tumors were evaluated at 20 wk after urethane injection. (B): H&E staining and immunohistochemistry of C/EBPα and Ki-67. H&E staining of on-Dox mice lung tissues with large tumors revealed C/EBPα-negative and strongly Ki-67 positive tumor cells. (C): The number and the size of the tumors and tumor burden were significantly higher in on-Dox mice than in no-Dox mice (n = 10/group, *p<0.01). (D): Mitotic index in the tumor of on-Dox and off-Dox mice (n = 7/group). The tumors in on-Dox mice exhibited greater proliferation than in no-Dox mice (*p<0.01).

To study how absence of C/EBPα affects tumor development, C/EBPα was deleted from the lung at 8 wk after urethane injection by administrating doxycycline at 14 wk of age for a total of 3 weeks (Figure 3A). Three weeks of doxycycline treatment was required to significantly delete C/EBPα from the lung of *Cebpa^flox/flox^* mice (Figure 3B). Lung tumors were evaluated at 20 wk after urethane injection (9 wk after C/EBPα deletion). Late-deletion of C/EBPα significantly increased the number and size of lung tumors and the tumor burden in on-Dox *Cebpα^Δ/Δ^* mice compared to no-Dox *Cebpa^flox/flox^* mice (Figure 3C). Tumor proliferation was also significantly enhanced by C/EBPα deletion (Figure 3B, D). Due to the shorter period of C/EBPα deletion, the tumor size and tumor burden in the late-deletion model were both smaller than those in the early-deletion model at 20 wk after urethane injection (Figure 1B). Interestingly, doxycycline administration rapidly increased the number of tumors in the late-deletion model and the number of tumors at 20 wk was similar to that of the early-deletion model (the number of tumors; late vs. early: 14.4±1.7 vs. 11.5±1.7, p = 0.25). These results indicated that loss of C/EBPα promotes tumor promotion and progression rather than tumor initiation.

### C/EBPα Expression in Tumor was Lost during Tumor Progression

**Figure 4:**
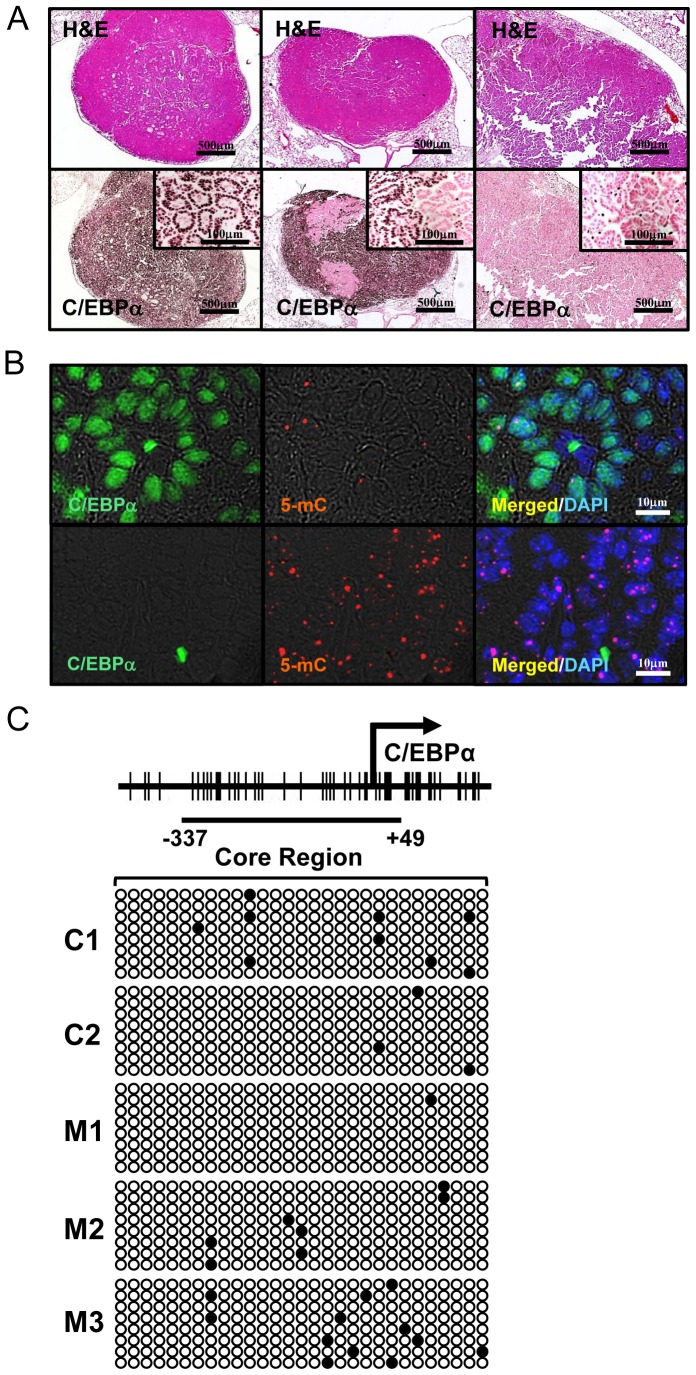
Natural C/EBPα Silencing and Promoter DNA Methylation in Urethane-Induced Tumors. (A): H&E staining and C/EBPα immunohistochemistry in urethane induced tumors in control mice evaluated 60 wk after urethane injection. *Left column*: C/EBPα-entirely positive tumor, *Middle column*: C/EBPα-partially positive tumor and *Right column*: C/EBPα-entirely negative tumor. The samples in each row were taken from serial sections. At least one partially positive tumor was observed in all mice (26/26). Entirely negative tumors were observed in 6 out of 26 mice. (B): Double immunofluorescence of C/EBPα and 5-mC in entirely-positive and entirely-negative tumor in long-observed model. C/EBPα-positive cells were weak or negative for 5-mC (upper row), while C/EBPα-negative cells stained positive for 5-mC (lower row), suggesting that C/EBPα-negative cells revealed DNA hypermethylation. (C): Bisulfite sequencing analysis for 3 entirely C/EBPα-negative tumors (M1, M2 and M3) and 2 C/EBPα entirely positive tumors (C1 and C2, 28 wk after urethane injection). *Arrow*, transcription start site; the diagrams of the core-promoter sites (−337 to +49) are drawn to scale. Promoter DNA methylation was weak in entirely C/EBPα-negative tumors. Each row represents an individual clone. *White circles, unmethylated CG dinucleotides; black circles, methylated CG dinucleotides*.

Although C/EBPα was strongly induced in all tumors in control mice at 28 wk after urethane injection, we found that this expression became weakened or absent as tumors progressed, similar results observed in human NSCLC [10], [11]. To clarify the mechanisms of decreased C/EBPα in progressed lung tumors, urethane injected control mice (at 6 wk of age) were followed until more than 10% of their body weight at 6 wk of age was lost. Average date of euthanasia was 63.7±1.2 wk after urethane injection. All of the 26 control mice had lung adenomas and/or adenocarcinomas evaluated by H&E staining (Figure 4A, upper panels). Immunohistochemistry of C/EBPα demonstrated 3 staining patterns: 1) entirely-positive, 2) partially-negative and 3) entirely-negative in lung tumors (Figure 4A, lower panels). Partially-negative C/EBPα tumors were found in all of the 26 mice, while 6 out of 26 mice had C/EBPα entirely-negative tumors.

### Loss of C/EBPα in Tumors is not by Aberrant Promoter DNA Methylation in Mice

To investigate the relation between C/EBPα expression and DNA methylation, tumors were stained with 5-mC. C/EBPα-negative cells in tumors were highly methylated compared to C/EBPα positive tumor cells detected by immunofluorescence of 5-mC (Figure 4B). Our findings suggest that C/EBPα expression might be lost from lung tumor cells by aberrant promoter DNA methylation as previously shown in human NSCLC [12]. Because methylation is more critical in the core region than in non-core region for DNA silencing in cancer cells [21], [22], the methylation of the core region in the Cebpa gene sequence was evaluated using isolated C/EBPα entirely-negative tumors. C/EBPα entirely-positive tumor tissues isolated from control mice at 28 wk after urethane injection were used for comparison. Contrary to our assumption, our mouse model represented no specific aberrant DNA methylation in isolated C/EBPα entirely-negative tumors (Figure 4C). Thus, there was no relationship between aberrant DNA methylation of core region and C/EBPα absence in tumor.

### C/EBPα is Induced by p38 MAP Kinase Activation in Lung Tumors

**Figure 5:**
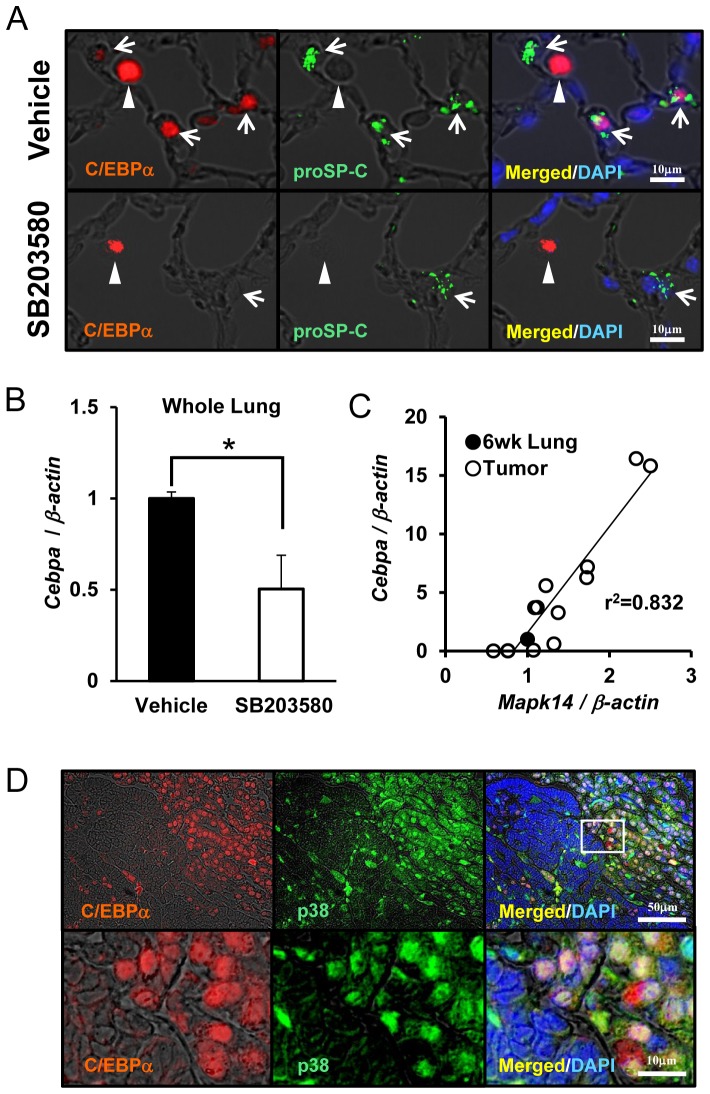
Regulation of C/EBPα through p38 MAP kinase. (A): Double immunofluorescence of C/EBPα and proSP-C. Alveolar type II cells and alveolar macrophages in vehicle-injected mice were positive for C/EBPα expression, while p38 MAP kinase inhibitor, SB203580, suppressed C/EBPα expression only in alveolar type II cells. Arrow head: alveolar macrophage. Arrow: alveolar type II cells. (B): qRT-PCR analysis of *Cebpa* mRNA expression. SB203580 significantly suppressed *Cebpa* mRNA expression in whole lung samples (n = 3, *p<0.05). C/EBPα expression is regulated by p38 MAP kinase *in vivo*. ((C): qRT-PCR for tumor tissue gene expression. The data were normalized to the expression in whole lung tissues from 6 wk old mice (n = 4, solid circles). Open circles represent tumor samples. *Cebpa* and *MAPK14* mRNA expressions in tumors were significantly correlated (n = 13, r^2^ = 0.832, p<0.0001, *Spearman's* correlation). (D): Double immunofluorescence of C/EBPα and p38 in C/EBPα partially positive tumors. In white rectangle in upper row, a high magnification clearly shows double-positive or –negative for C/EBPα and p38 expressions in tumor cells.

The strong C/EBPα expression in lung tumors was lost through an unknown mechanism as the tumors progressed. To determine if p38 MAP kinase regulates C/EBPα expression in normal lung, the p38 MAP kinase inhibitor (SB203580) was intraperitoneally injected into control mice and C/EBPα expression was determined by immunohistochemistry. As shown in Figure 5A and 5B, SB203580 successfully inhibited C/EBPα in alveolar type II cells and significantly inhibited *Cebpa* mRNA expression in the lungs. These data demonstrate that alveolar epithelial C/EBPα is regulated by p38 MAP kinase *in vivo*.

To investigate the mechanism of extinguished C/EBPα expression in tumors of long-observed model, total RNA was isolated from paraffin-embedded adenocarcinoma tissue from 13 urethane-injected mice followed by analysis of *Cebpa* and *MAPK14* by qRT-PCR. Although MAPK14 (p38α) is a known regulator of C/EBPα expression in lung stem cells during cell differentiation [16], [23], its role in lung tumor cells are unknown. We found that *Cebpa* mRNA expression strongly correlated with *MAPK14* mRNA in tumors (r^2^ = 0.832, p<0.0001, *Spearman's* correlation) (Figure 5C). As demonstrated by double immunofluorescence of C/EBPα and p38 MAP kinase, the majority of the tumor cells were either double positive or double negative (Figure 5DC). Collectively, these data suggest that the p38α MAP kinase pathway is a key regulator of C/EBPα expression in urethane-induced tumors and that decreased or absence of p38α MAP kinase in adenocarcinoma regulates C/EBPα expression in tumors.

## Discussion

In the present study, we demonstrated that decreased C/EBPα expression in the lung epithelial cells does not initiate lung tumor, but enhances tumor promotion and progression which results in short survival in mice. No differences for spontaneous tumor occurrence were detected after the long term C/EBPα deletion in the lung. Therefore, the deletion of C/EBPα is not oncogenic. Although C/EBPα expression was dispensable for lung under the normal condition, C/EBPα was strongly induced in urethane-induced tumors and suppressed tumor promotion/progression by regulating cell proliferation. This is the first study to directly demonstrate that C/EBPα exhibits lung tumor suppressor activity *in vivo*.

Frequency of spontaneous tumors and susceptibility to urethane differ between mouse strains. Urethane is metabolized by CYP2E1 to epoxide, which interacts with DNA to form carcinogenic DNA adducts [24]. CYP2E1 and NFκB activities determine susceptibility to urethane in mice [17], [24]. Although FVB/N mouse has higher susceptibility and C57BL6/J has less susceptible to urethane, our mice showed consistent susceptibility for tumor formation, suggesting a homogeneous mixed-strain background.

C/EBPα has been shown to have a critical role in the lung under abnormal conditions such as lung injury and repair. Our previous studies highlighted the critical cytoprotective functions of C/EBPα in the lung and indicated that it may suppress tumor development. C/EBPα protects the lung from hyperoxia by regulating surfactant synthesis and oxidative sensor [8], suggesting that changes in the redox status might affect tumor development in mice. C/EBPα also regulates the protease/anti-protease balance in Clara cells during bronchiolar injury and repair [25]. *Cebpα^Δ/Δ^* mice lack lympho-epithelial Kazal-type-related inhibitor (LEKTI, coded by *Spink5*), which is a serine protease inhibitor that strongly inhibits kallikrein-related peptidase (KLK) activity [9], [26]. Although we have not evaluated the activity of LEKTI in tumors, *Spink5* mRNA was significantly up-regulated in control mice at 28 wk after urethane injection, but unchanged in *Cebpα^Δ/Δ^* mice (Figure S2). The tumor derived KLKs promote tumor cell migration by activating PAR1 [27], [28], therefore, LEKTI might be a potential functional downstream target of C/EBPα.

The tumor suppressor function of C/EBPα has been well documented in non-lung tissues, particularly in the liver, skin and myeloid malignancies [29]–[31]. In our study, tumors in *Cebpα^Δ/Δ^* mice showed increased proliferation determined by increased Ki-67 and mitosis-specific pH 3 expression. The cell cycle regulatory function of C/EBPα has been demonstrated in non-lung cells by interacting with p21 [13], E2Fs [32], [33], Cdk2 [34], Cdk4 [34], SWI/SNF complex [35], Rb family members [36], and 1, 25^−^dihydroxyvitamin D3 [13], [32]–[37], but its role in lung cells remains poorly understood. Unlike hematopoietic cells, the human lung cancer cell line H358 did not exhibit any changes in the cell cycle upon transfection with CEBPA, and the CEBPA transgene stimulated apoptosis only in the context of toxic stimulation by zinc [3], [11]. We evaluated the inhibition of *CEBPA* by small interfering RNA in A549 cells, but cell proliferation evaluated by XTT assay was same as control cells (Data not shown). Since C/EBPα has an emergent role, urethane-induced inflammation might modify C/EBPα by the mechanisms such as phosphorylation, sumoylation or binding to co-factors as previously reported [38]–[42].

The present study demonstrated that p38 MAP kinase activation is required for C/EBPα expression in alveolar epithelial cells and that *Mapk14* expression was strongly correlated with *Cebpa* expression in tumors. The interaction between the two factors has been studied for cell differentiation in non-lung cells including keratinocyte, hepatocyte and neutrophils [43]–[45]. Hui et al. reported that *MAPK14^Δ/Δ^* mice died before postnatal day 4 due to respiratory distress with impaired ATII cell maturation [46], which is the same phenotype observed when Cebpa was deleted embryonically [7]. In another study, the *K-Ras^G12V^*-induced tumor model intercrossed with *MAPK14^Δ/Δ^* mice (*MAPK14* deletion in adult) demonstrated high tumor frequency with low expression of C/EBPα and FoxA2 in the lung [16]. This study also demonstrated the down-regulation of p38α in human NSCLC [16], consistent with the results in our long-observed study. These two studies focused on lung stem cell or cancer stem cell differentiation in response to p38α MAP kinase pathway and claimed that p38α MAP kinase inactivity by using p38 MAP kinase inhibitors impaired lung stem cell differentiation and increased the tumor susceptibility [16], [23]. In the present study, we demonstrated that p38 MAP kinase regulates C/EBPα in normal lung epithelial cells and that C/EBPα expression in tumors was silenced as tumors progressed in long-observed model. Furthermore, CC10^+^ and SP-C^+^ double positive putative stem cells were rarely observed in urethane-induced tumors, either in *Cebpα^Δ/Δ^* mice or long-observed control mice group (data not shown), suggesting that the tumor progression in *Cebpα^Δ/Δ^* mice might not be associated with lung stem cell expansion. In long-observed model, we could not find the difference in survival time after urethane-injection and mitotic index in tumor cells between having and not having C/EBPα negative tumor in mice (data not shown), suggesting that C/EBPα silencing is not an only factor determining the lifetime risk due to lung malignancy, which is consistent with the previous study for NSCLC [10].

p38 MAP kinase inhibitors have been under the clinical trials for cardiovascular disease and chronic obstructive lung disease, and one of p38 MAP kinase inhibitors demonstrated significant anti-inflammatory effects by ameliorating disease biomarkers [47], [48]. Although we showed C/EBPα silencing is not oncogenic in the lung, a potential risk of C/EBPα silencing in the lung by treatment with p38 MAP kinase inhibitors should be given a caution and these patients require screening for lung cancer. A dose threshold of p38 MAP kinase inhibitor for anti-inflammation and the silencing of C/EBPα and p38α expression should be evaluated to avoid lung cancer progression.

FoxA2, also regulated by p38α MAP kinase, is downregulated in NSCLC and is a candidate lung tumor suppressor [49]. The induction of FoxA2 in H358 cells has been shown to result in proliferation arrest and apoptosis *in vitro* [50]. Although FoxA2 is described as a downstream target of C/EBPα in the fetal lung and *in vitro* [7], [50], FoxA2 was strongly expressed in the urethane-induced tumors of *Cebpα^Δ/Δ^* mice (Figure S3A) and *Foxa2* mRNA was also induced in the tumors from *Cebpα^Δ/Δ^* mice (Figure S3B), suggesting that FoxA2 does not suppress urethane-injected tumor in *Cebpα^Δ/Δ^* mice and is not a downstream target of C/EBPα in adult mice.

The mechanisms of C/EBPα absence in NSCLC have been demonstrated by using lung cancer cell lines. TRIB2 was demonstrated as a suppressor of C/EBPα in human NSCLC [15], but *Trib2* mRNA expression in urethane-induced tumors of our long-observed model was below the detection limit compared to normal lung tissue (Data not shown). In spite of high 5-mC expression in C/EBPα negative tumor cells, our data showed that core region of Cebpa was not strongly methylated. Since *Mapk14* expression was downregulated similarly as *Cebpa* expression in tumors, we assumed that signal upstream of MAP kinase14 might be silenced by DNA methylation. One limitation of this study is the use of a mouse model. In humans, the CEBPA gene is located on chromosome 19, whereas in mice, the Cebpa gene is located on chromosome 7. Furthermore, the promoter sequence is not identical between humans and mice. Thus, susceptibility for the promoter DNA methylation might be different between species. Our study demonstrated that C/EBPα absence was not due to the aberrant DNA methylation of the core promoter region in a murine model, but promoter hypermethylation cannot be ruled out as a mechanism of C/EBPα silencing in human tumors.

In summary, the present study demonstrated a cell specific role of C/EBPα as a lung tumor suppressor *in vivo*. C/EBPα suppressed tumor promotion/progression in a urethane-induced tumor model by regulating cell proliferation. C/EBPα expression in lung epithelial cells was regulated by p38α MAP kinase and p38 MAP kinase inactivation resulted in absence of C/EBPα expression. The p38α MAP kinase pathway is critical for tumor suppression and C/EBPα is one of the effective downstream targets of this pathway. Re-activation of the p38α MAP kinase pathway might be a useful therapy for lung cancer.

## Supporting Information

Figure S1 **Lung epithelial C/EBPα deletion in** ***Cebpα^Δ/Δ^*** **mice.** (A): Triple transgenic system for the lung epithelial specific deletion of C/EBPα by doxycycline administration. By using *Scgb1a1* promoter, *Cre* was expressed in lung epithelial cells. The targeting construct deletion was mediated by Cre/LoxP system. (B): *Cebpa* expression by qRT-PCR in whole lungs and isolated type II cells. *Cebpa* expression is significantly lower in *Cebpα^Δ/Δ^* mice in both lungs and isolated ATII cells (*p<0.01, n = 4/group).
(TIF)

Figure S2 **mRNA expression of** ***Spink5*** **in whole lungs.** In control mice, *Spink5* expression at 28 wk was significantly higher in urethane-injected mice than saline-injected mice (*p<0.05, n = 4/group).
(TIF)

Figure S3 **Significant FoxA2 expression in** ***Cebpα^Δ/Δ^*** **mice.** (A): Double immunofluorescence of C/EBPα and FoxA2 in lung tumors at 28 wk after urethane injection. In control mice, both C/EBPα and FoxA2 were expressed in tumor. Although C/EBPα was absent in the tumors of *Cebpα^Δ/Δ^* mice, FoxA2 was strongly expressed in tumors, suggesting that FoxA2 expression is independent of C/EBPα. Tu: tumor (B): *Foxa2* mRNA expression in whole lungs was significantly up-regulated in both control and *Cebpα^Δ/Δ^* mice 28 wk after urethane injection (n = 4/group, *p<0.05). This expression was significantly stronger in tumor tissues than in non-tumor tissues in both control and *Cebpα^Δ/Δ^* mice (n = 4/group, *p<0.05).
(TIF)

## References

[1] Biesalski HK, Bueno de Mesquita B, Chesson A, Chytil F, Grimble R, et al (1998) European Consensus Statement on Lung Cancer: risk factors and prevention. Lung Cancer Panel CA: a cancer journal for clinicians 48: 167–176 discussion 164–166. 9594919 10.3322/canjclin.48.3.167

[2] Fong KM, Sekido Y, Gazdar AF, Minna JD (2003) Lung cancer. 9: Molecular biology of lung cancer: clinical implications. Thorax 58: 892–900. 14514947 10.1136/thorax.58.10.892 PMC1746489

[3] Schuster MB, Porse BT (2006) C/EBPalpha: a tumour suppressor in multiple tissues? Biochim Biophys Acta 1766: 88–103. 16616425 10.1016/j.bbcan.2006.02.003

[4] Lopez RG, Garcia-Silva S, Moore SJ, Bereshchenko O, Martinez-Cruz AB, et al (2009) C/EBPalpha and beta couple interfollicular keratinocyte proliferation arrest to commitment and terminal differentiation. Nat Cell Biol 11: 1181–1190. 19749746 10.1038/ncb1960

[5] Ramji DP, Foka P (2002) CCAAT/enhancer-binding proteins: structure, function and regulation. Biochem J 365: 561–575. 12006103 10.1042/BJ20020508 PMC1222736

[6] Nord M, Cassel TN, Braun H, Suske G (2000) Regulation of the Clara cell secretory protein/uteroglobin promoter in lung. Ann N Y Acad Sci 923: 154–165. 11193754 10.1111/j.1749-6632.2000.tb05527.x

[7] Martis PC, Whitsett JA, Xu Y, Perl AK, Wan H, et al (2006) C/EBP{alpha} is required for lung maturation at birth. Development 133: 1155–1164. 16467360 10.1242/dev.02273

[8] Xu Y, Saegusa C, Schehr A, Grant S, Whitsett JA, et al (2009) C/EBP{alpha} is required for pulmonary cytoprotection during hyperoxia. Am J Physiol Lung Cell Mol Physiol 297: L286–298. 19465518 10.1152/ajplung.00094.2009 PMC2742785

[9] Sato A, Xu Y, Whitsett JA, Ikegami M (2012) C/EBPalpha Regulates the Protease/Anti-protease Balance Required for Bronchiolar Epithelium Regeneration. Am J Respir Cell Mol Biol 10.1165/rcmb.2011-0239OC PMC3488626 22652201

[10] Costa DB, Li S, Kocher O, Feins RH, Keller SM, et al (2007) Immunohistochemical analysis of C/EBPalpha in non-small cell lung cancer reveals frequent down-regulation in stage II and IIIA tumors: a correlative study of E3590. Lung Cancer 56: 97–103. 17239984 10.1016/j.lungcan.2006.11.023 PMC3380244

[11] Halmos B, Huettner CS, Kocher O, Ferenczi K, Karp DD, et al (2002) Down-regulation and antiproliferative role of C/EBPalpha in lung cancer. Cancer Res 62: 528–534. 11809705

[12] Tada Y, Brena RM, Hackanson B, Morrison C, Otterson GA, et al (2006) Epigenetic modulation of tumor suppressor CCAAT/enhancer binding protein alpha activity in lung cancer. J Natl Cancer Inst 98: 396–406. 16537832 10.1093/jnci/djj093

[13] Timchenko NA, Harris TE, Wilde M, Bilyeu TA, Burgess-Beusse BL, et al (1997) CCAAT/enhancer binding protein alpha regulates p21 protein and hepatocyte proliferation in newborn mice. Mol Cell Biol 17: 7353–7361. 9372966 10.1128/mcb.17.12.7353 PMC232591

[14] Costa DB, Dayaram T, D'Alo F, Wouters BJ, Tenen DG, et al (2006) C/EBP alpha mutations in lung cancer. Lung Cancer 53: 253–254. 16765476 10.1016/j.lungcan.2006.04.011

[15] Grandinetti KB, Stevens TA, Ha S, Salamone RJ, Walker JR, et al (2011) Overexpression of TRIB2 in human lung cancers contributes to tumorigenesis through downregulation of C/EBPalpha. Oncogene 30: 3328–3335. 21399661 10.1038/onc.2011.57 PMC3382061

[16] Ventura JJ, Tenbaum S, Perdiguero E, Huth M, Guerra C, et al (2007) p38alpha MAP kinase is essential in lung stem and progenitor cell proliferation and differentiation. Nat Genet 39: 750–758. 17468755 10.1038/ng2037

[17] Stathopoulos GT, Sherrill TP, Cheng DS, Scoggins RM, Han W, et al (2007) Epithelial NF-kappaB activation promotes urethane-induced lung carcinogenesis. Proc Natl Acad Sci U S A 104: 18514–18519. 18000061 10.1073/pnas.0705316104 PMC2141808

[18] Nikitin AY, Alcaraz A, Anver MR, Bronson RT, Cardiff RD, et al (2004) Classification of proliferative pulmonary lesions of the mouse: recommendations of the mouse models of human cancers consortium. Cancer Res 64: 2307–2316. 15059877 10.1158/0008-5472.can-03-3376

[19] Mahler JF, Stokes W, Mann PC, Takaoka M, Maronpot RR (1996) Spontaneous lesions in aging FVB/N mice. Toxicol Pathol 24: 710–716. 8994298 10.1177/019262339602400606

[20] Glaros S, Cirrincione GM, Palanca A, Metzger D, Reisman D (2008) Targeted knockout of BRG1 potentiates lung cancer development. Cancer Res 68: 3689–3696. 18483251 10.1158/0008-5472.CAN-07-6652

[21] Ushijima T, Okochi-Takada E (2005) Aberrant methylations in cancer cells: where do they come from? Cancer Sci 96: 206–211. 15819717 10.1111/j.1349-7006.2005.00035.x PMC11159910

[22] Ushijima T (2005) Detection and interpretation of altered methylation patterns in cancer cells. Nat Rev Cancer 5: 223–231. 15719030 10.1038/nrc1571

[23] Oeztuerk-Winder F, Guinot A, Ochalek A, Ventura JJ (2012) Regulation of human lung alveolar multipotent cells by a novel p38alpha MAPK/miR-17-92 axis. EMBO J 31: 3431–3441. 22828869 10.1038/emboj.2012.192 PMC3419929

[24] Forkert PG (2010) Mechanisms of lung tumorigenesis by ethyl carbamate and vinyl carbamate. Drug Metab Rev 42: 355–378. 20205516 10.3109/03602531003611915

[25] Sato A, Xu Y, Whitsett JA, Ikegami M (2012) CCAAT/Enhancer Binding Protein-alpha Regulates the Protease/Antiprotease Balance Required for Bronchiolar Epithelium Regeneration. Am J Respir Cell Mol Biol 47: 454–463. 22652201 10.1165/rcmb.2011-0239OC PMC3488626

[26] Magert HJ, Standker L, Kreutzmann P, Zucht HD, Reinecke M, et al (1999) LEKTI, a novel 15-domain type of human serine proteinase inhibitor. J Biol Chem 274: 21499–21502. 10419450 10.1074/jbc.274.31.21499

[27] Oikonomopoulou K, Diamandis EP, Hollenberg MD (2010) Kallikrein-related peptidases: proteolysis and signaling in cancer, the new frontier. Biol Chem 391: 299–310. 20180639 10.1515/BC.2010.038

[28] Cisowski J, O'Callaghan K, Kuliopulos A, Yang J, Nguyen N, et al (2011) Targeting protease-activated receptor-1 with cell-penetrating pepducins in lung cancer. Am J Pathol 179: 513–523. 21703428 10.1016/j.ajpath.2011.03.025 PMC3123854

[29] Shim M, Powers KL, Ewing SJ, Zhu S, Smart RC (2005) Diminished expression of C/EBPalpha in skin carcinomas is linked to oncogenic Ras and reexpression of C/EBPalpha in carcinoma cells inhibits proliferation. Cancer Res 65: 861–867. 15705884

[30] Jin J, Wang GL, Timchenko L, Timchenko NA (2009) GSK3beta and aging liver. Aging 1: 582–585. 20157540 10.18632/aging.100060 PMC2806031

[31] Timchenko NA (2009) Aging and liver regeneration. Trends in endocrinology and metabolism: TEM 20: 171–176. 19359195 10.1016/j.tem.2009.01.005

[32] Loomis KD, Zhu S, Yoon K, Johnson PF, Smart RC (2007) Genetic ablation of CCAAT/enhancer binding protein alpha in epidermis reveals its role in suppression of epithelial tumorigenesis. Cancer Res 67: 6768–6776. 17638888 10.1158/0008-5472.CAN-07-0139 PMC3773581

[33] Slomiany BA, D'Arigo KL, Kelly MM, Kurtz DT (2000) C/EBPalpha inhibits cell growth via direct repression of E2F-DP-mediated transcription. Mol Cell Biol 20: 5986–5997. 10913181 10.1128/mcb.20.16.5986-5997.2000 PMC86075

[34] Wang H, Iakova P, Wilde M, Welm A, Goode T, et al (2001) C/EBPalpha arrests cell proliferation through direct inhibition of Cdk2 and Cdk4. Mol Cell 8: 817–828. 11684017 10.1016/s1097-2765(01)00366-5

[35] Muller C, Calkhoven CF, Sha X, Leutz A (2004) The CCAAT enhancer-binding protein alpha (C/EBPalpha) requires a SWI/SNF complex for proliferation arrest. The Journal of biological chemistry 279: 7353–7358. 14660596 10.1074/jbc.M312709200

[36] Timchenko NA, Wilde M, Darlington GJ (1999) C/EBPalpha regulates formation of S-phase-specific E2F-p107 complexes in livers of newborn mice. Molecular and cellular biology 19: 2936–2945. 10082561 10.1128/mcb.19.4.2936 PMC84088

[37] Dhawan P, Wieder R, Christakos S (2009) CCAAT enhancer-binding protein alpha is a molecular target of 1,25-dihydroxyvitamin D3 in MCF-7 breast cancer cells. The Journal of biological chemistry 284: 3086–3095. 19054766 10.1074/jbc.M803602200 PMC2631956

[38] Muller C, Calkhoven CF, Sha X, Leutz A (2004) The CCAAT enhancer-binding protein alpha (C/EBPalpha) requires a SWI/SNF complex for proliferation arrest. J Biol Chem 279: 7353–7358. 14660596 10.1074/jbc.M312709200

[39] Iakova P, Awad SS, Timchenko NA (2003) Aging reduces proliferative capacities of liver by switching pathways of C/EBPalpha growth arrest. Cell 113: 495–506. 12757710 10.1016/s0092-8674(03)00318-0

[40] Wang GL, Timchenko NA (2005) Dephosphorylated C/EBPalpha accelerates cell proliferation through sequestering retinoblastoma protein. Mol Cell Biol 25: 1325–1338. 15684384 10.1128/MCB.25.4.1325-1338.2005 PMC548025

[41] Wang GL, Salisbury E, Shi X, Timchenko L, Medrano EE, et al (2008) HDAC1 cooperates with C/EBPalpha in the inhibition of liver proliferation in old mice. J Biol Chem 283: 26169–26178. 18622015 10.1074/jbc.M803544200 PMC2533775

[42] Khanna-Gupta A (2008) Sumoylation and the function of CCAAT enhancer binding protein alpha (C/EBP alpha). Blood Cells Mol Dis 41: 77–81. 18406180 10.1016/j.bcmd.2008.02.011 PMC2505045

[43] Efimova T, Deucher A, Kuroki T, Ohba M, Eckert RL (2002) Novel protein kinase C isoforms regulate human keratinocyte differentiation by activating a p38 delta mitogen-activated protein kinase cascade that targets CCAAT/enhancer-binding protein alpha. J Biol Chem 277: 31753–31760. 12080077 10.1074/jbc.M205098200

[44] Geest CR, Buitenhuis M, Laarhoven AG, Bierings MB, Bruin MC, et al (2009) p38 MAP kinase inhibits neutrophil development through phosphorylation of C/EBPalpha on serine 21. Stem Cells 27: 2271–2282. 19544470 10.1002/stem.152

[45] Qiao L, MacDougald OA, Shao J (2006) CCAAT/enhancer-binding protein alpha mediates induction of hepatic phosphoenolpyruvate carboxykinase by p38 mitogen-activated protein kinase. J Biol Chem 281: 24390–24397. 16807249 10.1074/jbc.M603038200

[46] Hui L, Bakiri L, Mairhorfer A, Schweifer N, Haslinger C, et al (2007) p38alpha suppresses normal and cancer cell proliferation by antagonizing the JNK-c-Jun pathway. Nat Genet 39: 741–749. 17468757 10.1038/ng2033

[47] Cheriyan J, Webb AJ, Sarov-Blat L, Elkhawad M, Wallace SM, et al (2011) Inhibition of p38 mitogen-activated protein kinase improves nitric oxide-mediated vasodilatation and reduces inflammation in hypercholesterolemia. Circulation 123: 515–523. 21262998 10.1161/CIRCULATIONAHA.110.971986

[48] Lomas DA, Lipson DA, Miller BE, Willits L, Keene O, et al (2012) An oral inhibitor of p38 MAP kinase reduces plasma fibrinogen in patients with chronic obstructive pulmonary disease. J Clin Pharmacol 52: 416–424. 22090363 10.1177/0091270010397050

[49] Basseres DS, D'Alo F, Yeap BY, Lowenberg EC, Gonzalez DA, et al (2012) Frequent downregulation of the transcription factor Foxa2 in lung cancer through epigenetic silencing. Lung Cancer 77: 31–37. 22341411 10.1016/j.lungcan.2012.01.011 PMC3368092

[50] Halmos B, Basseres DS, Monti S, D'Alo F, Dayaram T, et al (2004) A transcriptional profiling study of CCAAT/enhancer binding protein targets identifies hepatocyte nuclear factor 3 beta as a novel tumor suppressor in lung cancer. Cancer Res 64: 4137–4147. 15205324 10.1158/0008-5472.CAN-03-4052

