# Supplementary figures and images for: CCAAT/Enhancer-Binding Protein-α Suppresses Lung Tumor Development in Mice through the p38α MAP Kinase Pathway
Source: PLoS One. 2013 Feb 20;8(2):e57013. doi: 10.1371/journal.pone.0057013 (PMC3577786; doi:10.1371/journal.pone.0057013)

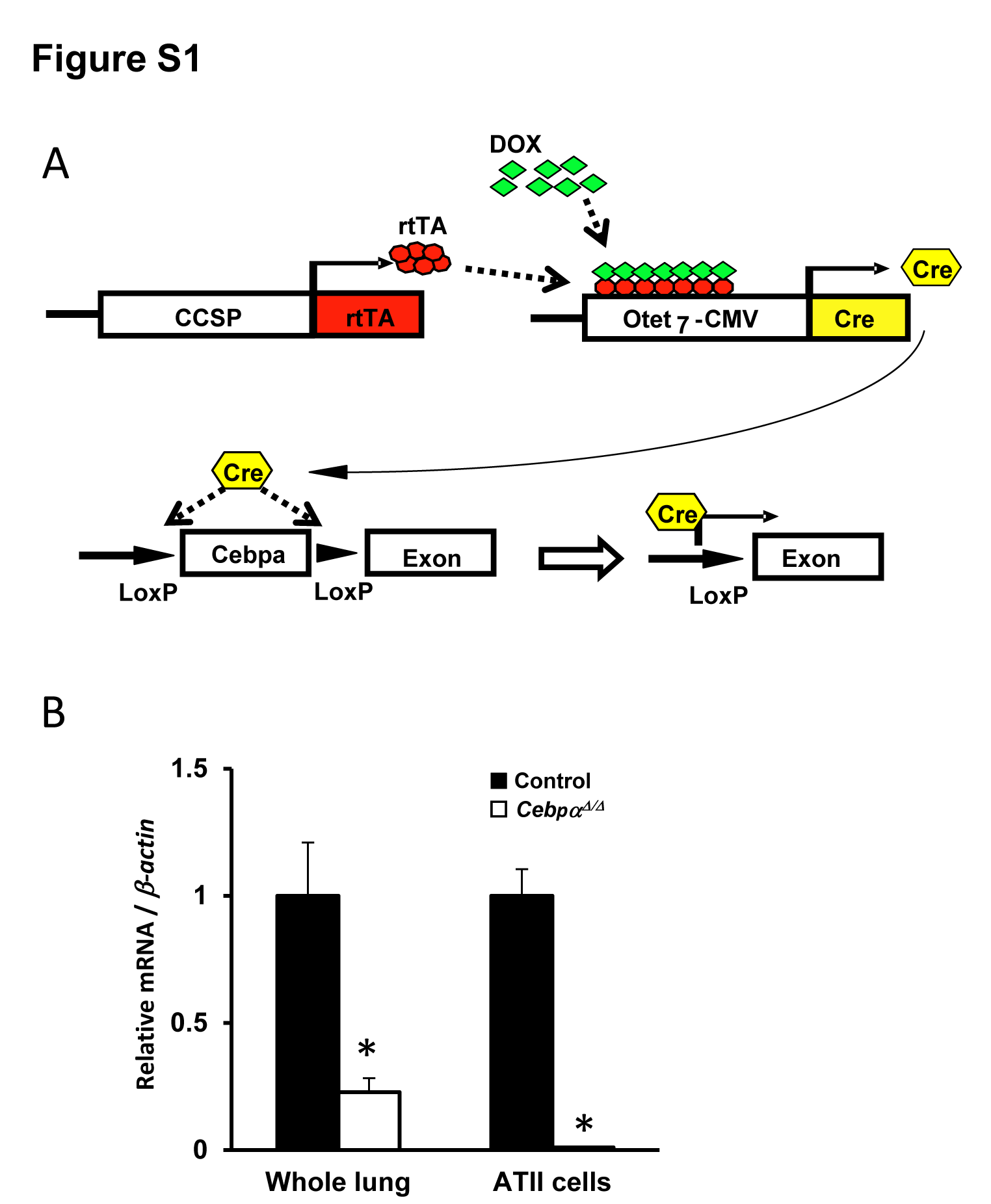

Supplement: Figure S1 — Lung epithelial C/EBPα deletion in CebpαΔ/Δ mice. (A): Triple transgenic system for the lung epithelial specific deletion of C/EBPα by doxycycline administration. By using Scgb1a1 promoter, Cre was expressed in lung epithelial cells. The targeting construct deletion was mediated by Cre/LoxP system. (B): Cebpa expression by qRT-PCR in whole lungs and isolated type II cells. Cebpa expression is significantly lower in CebpαΔ/Δ mice in both lungs and isolated ATII cells (*p<0.01, n = 4/group). (TIF) [file pone.0057013.s001.tif]

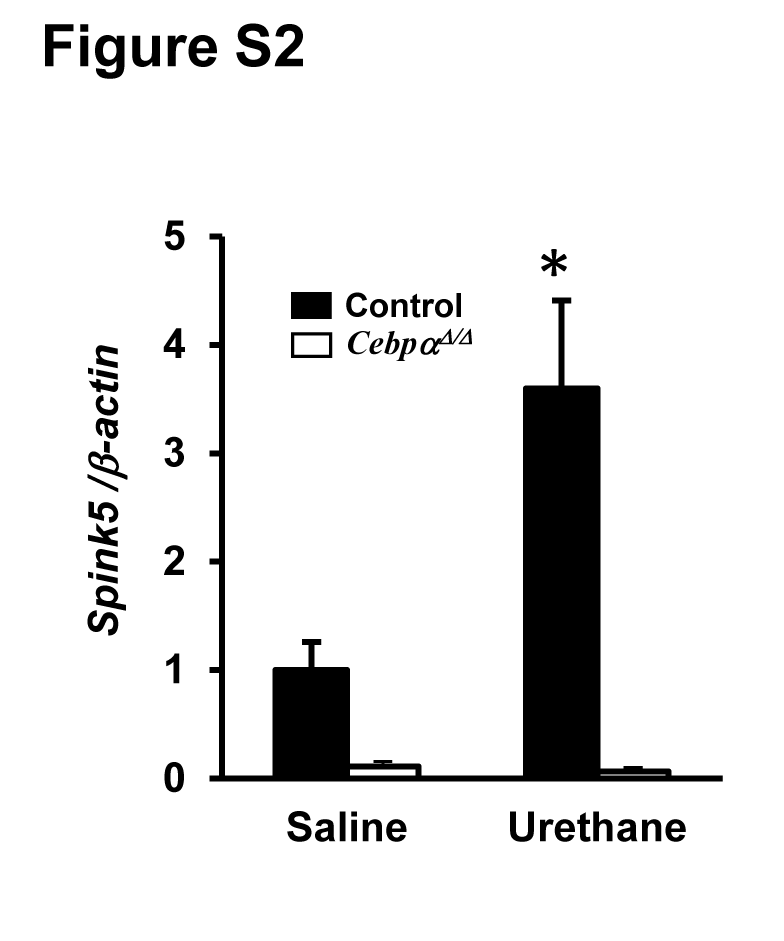

Supplement: Figure S2 — mRNA expression of Spink5 in whole lungs. In control mice, Spink5 expression at 28 wk was significantly higher in urethane-injected mice than saline-injected mice (*p<0.05, n = 4/group). (TIF) [file pone.0057013.s002.tif]

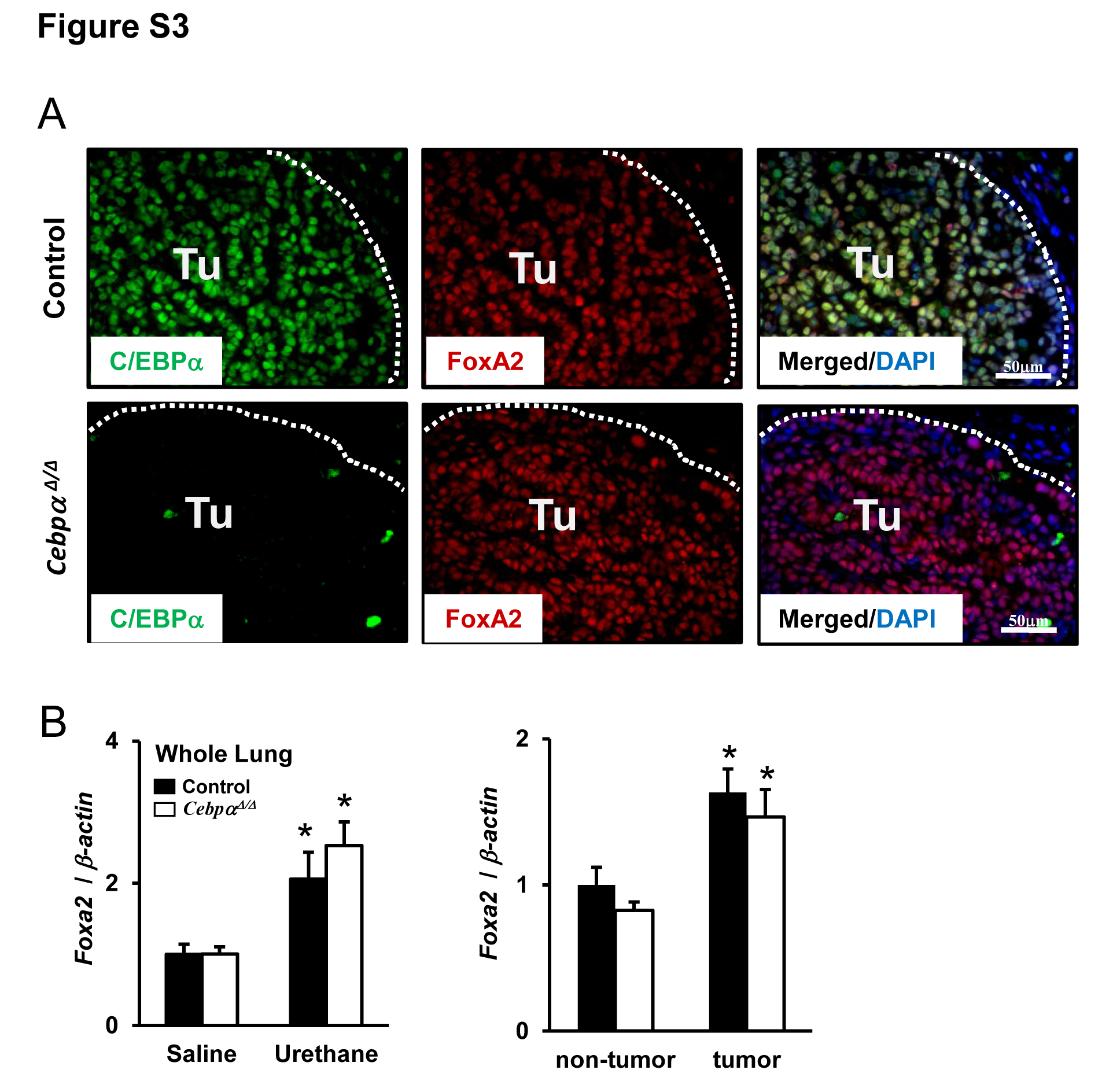

Supplement: Figure S3 — Significant FoxA2 expression in CebpαΔ/Δ mice. (A): Double immunofluorescence of C/EBPα and FoxA2 in lung tumors at 28 wk after urethane injection. In control mice, both C/EBPα and FoxA2 were expressed in tumor. Although C/EBPα was absent in the tumors of CebpαΔ/Δ mice, FoxA2 was strongly expressed in tumors, suggesting that FoxA2 expression is independent of C/EBPα. Tu: tumor (B): Foxa2 mRNA expression in whole lungs was significantly up-regulated in both control and CebpαΔ/Δ mice 28 wk after urethane injection (n = 4/group, *p<0.05). This expression was significantly stronger in tumor tissues than in non-tumor tissues in both control and CebpαΔ/Δ mice (n = 4/group, *p<0.05). (TIF) [file pone.0057013.s003.tif]
